# Reversible adapting layer produces robust single-crystal electrocatalyst for oxygen evolution

**DOI:** 10.1038/ncomms9106

**Published:** 2015-08-28

**Authors:** Ching-Wei Tung, Ying-Ya Hsu, Yen-Ping Shen, Yixin Zheng, Ting-Shan Chan, Hwo-Shuenn Sheu, Yuan-Chung Cheng, Hao Ming Chen

**Affiliations:** 1 Department of Chemistry, National Taiwan University, Taipei 106, Taiwan; 2 Program for Science and Technology of Accelerator Light Source, National Chiao Tung University, Hsinchu 300, Taiwan; 3 National Synchrotron Radiation Research Center, Hsinchu 300, Taiwan

## Abstract

Electrochemically converting water into oxygen/hydrogen gas is ideal for high-density renewable energy storage in which robust electrocatalysts for efficient oxygen evolution play crucial roles. To date, however, electrocatalysts with long-term stability have remained elusive. Here we report that single-crystal Co_3_O_4_ nanocube underlay with a thin CoO layer results in a high-performance and high-stability electrocatalyst in oxygen evolution reaction. An *in situ* X-ray diffraction method is developed to observe a strong correlation between the initialization of the oxygen evolution and the formation of active metal oxyhydroxide phase. The lattice of skin layer adapts to the structure of the active phase, which enables a reversible facile structural change that facilitates the chemical reactions without breaking the scaffold of the electrocatalysts. The single-crystal nanocube electrode exhibits stable, continuous oxygen evolution for >1,000 h. This robust stability is attributed to the complementary nature of defect-free single-crystal electrocatalyst and the reversible adapting layer.

## Abstract (web summary)

There is extensive research into water-oxidation electrocatalysts which exhibit long-term stability. Here, the authors report a single-crystal cobalt oxide electrocatalyst displaying high activity and stability, and develop an *in situ* X-ray diffraction method to probe the structure–activity relationship.

## Graphical abstract

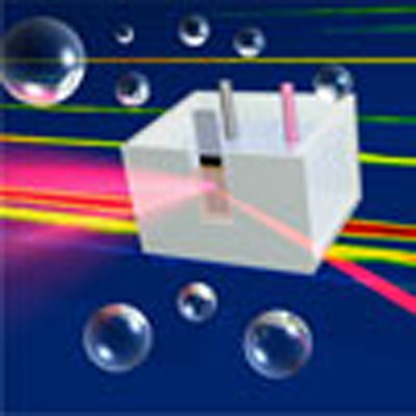

Growing concerns about global warming and increasing demands for energy have prompted the search for renewable energy sources and the development of improved energy storage options to replace fossil fuel technologies^1^,^2^,^3^,^4^,^5^,^6^,^7^. In many of the innovative approaches for addressing these challenges, photoelectrolyzing or electrolyzing water to produce oxygen and hydrogen gases is a highly scalable method for storing chemical fuels derived from renewable sources^8^,^9^,^10^. Therefore, the development of abundant, robust and efficient catalysts for photoelectrochemical/electrochemical production of oxygen are crucial for the long-term viability of our community.

In recent years, various materials including simple oxides^11^,^12^,^13^,^14^,^15^, combinations of transition metal oxides^16^,^17^,^18^,^19^,^20^, phosphates^21^,^22^, oxyhydroxide^23^,^24^, perovskites^10^,^25^ and nanocomposite materials^26^,^27^,^28^,^29^ have been used to induce an oxygen evolution reaction (OER) in alkaline environment. Despite the interesting catalytic behaviours of existing materials, an inexpensive and reliable electrocatalyst that does not develop defects to hinder charge transfer across the electrolyte/catalyst interface has yet to be achieved. Recent reports of highly economic and efficient electrochemical catalysts have significantly advanced this technology^14^, however the largest challenge concerning the reliability of the catalyst remains yet unconquered. A major reason for the slow progress of reliable catalysts in the OER is that the electrocatalyst bears most of the charge carriers during the harsh oxidization process because the OER normally occurs under a high anodic potential. A highly stable electrocatalyst has to transfer charge carriers effectively to the OER electrocatalyst/electrolyte interface yet retain a sufficient amount of oxidized species during anodization. To shed light on such complex surface reactions, we need a tool that allows *in situ* observation of the active phase of metal centres under anodization and a novel strategy to protect electrocatalysts under such harsh conditions in order to achieve reliable catalysts for OER.

The oxygen evolution activity of electrocatalysts depends strongly on corresponding surface structures and the adsorption energies of intermediates on metal oxide surfaces^30^,^31^. Generally speaking, surface structure can affect the activity of electrocatalysts and surface states can be further altered by introducing foreign elements, which leads to considerable decreases in overpotential and therefore increases in activity during the OER. In practical conditions, the reactions of OER are only involved within a several nanometre region of the catalyst surface, and *in situ* studies within this limited region are essential and extremely challenging. Recently, *in situ* Raman spectroscopy has been conducted to achieve this goal to reveal the intermediates through surface binding of OER reactants^11^. Moreover, Zhang *et al.*^32^ identified the surface bonding formation and relevant intermediates through time-resolved rapid-scan Fourier transform infrared spectroscopy, in which a surface superoxide species was revealed in a fast reaction cycle of OER. However, for designing efficient OER catalysts, *in situ* study for structure and crystallinity at the atomic level of the metal centres in the catalyst surface are vital and have yet to be achieved. The development of tools to study and investigate the surface of catalysts could provide a powerful means to elucidate fundamental processes in OER catalyst and eventually lead to a novel design principle.

Herein, we investigate single-crystal Co_3_O_4_ nanocubes with underlaying of uniform cobalt oxide (CoO) layers as OER electrocatalysts. The strategy is to incorporate a surface layer well adapted to the active phases of the catalytic metal centres to enhance the stability of the electrocatalysts. Furthermore, we develop an *in situ* grazing-angle X-ray diffraction method to track the atomic-scale structural changes on the surface of the electrode during OER in liquid conditions. This powerful technique reveals that the CoO layer can reversibly adapt to the harsh conditions of OER through reversible structural transformations, which results in a highly robust electrocatalyst. In addition, we choose Co_3_O_4_ because of its abundance, low cost, low electrical resistance, thermodynamic stability and moderate overpotential in neutral and alkaline conditions^33^. Using single-crystal nanocubes as OER electrocatalysts provide strong mechanical strength, minimal structural disorder and defect-free substrates. The structural defects in materials normally turn into trap states that play a dominant role in limiting carrier transportation, and effective carrier transportation within the substance determines the overall efficiency of the devices^34^,^35^. Accordingly, the low trap density of the single-crystal defect-free nanostructure enhances its carrier dynamics and thereby improve the electronic properties of the materials. The CoO layer on the surface of the Co_3_O_4_ nanocubes forms a reversible adaption junction, enabling a facile structural transformation^18^ for chemical reactions (for example, OER) and the formation of active phase without breaking the scaffold of the electrocatalysts (for example, Co_3_O_4_ nanocubes) to further reduce the overpotential.

## Results

### Structural characterization of Co_3_O_4_–CoO core–shell nanocube

**Figure 1:**
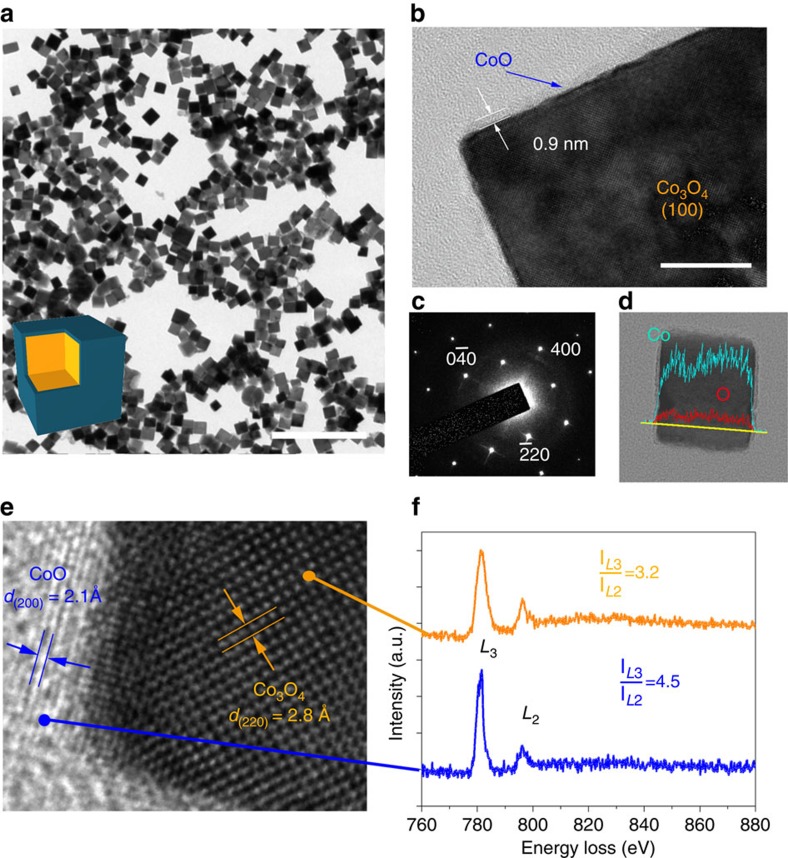
Structural characterization of the Co_3_O_4_@CoO single-crystal nanocubes. (**a**,**b**) A typical transmission electron micrograph. Scale bar, 200 nm (**a**); 10 nm (**b**). (**c**) Selected area electron diffraction pattern of an individual Co_3_O_4_@CoO single-crystal nanocube. (**d**) The corresponding elemental line scan from the Co and O of an individual Co_3_O_4_@CoO single-crystal nanocube. (**e**) HRTEM image of a Co_3_O_4_@CoO single-crystal nanocube. The dark area is the Co_3_O_4_ and the light area is the CoO. (**f**) Corresponding electron energy-loss spectrum of a Co_3_O_4_@CoO SC nanocube. The upper spectrum was recorded at the dark area of **e** and the lower spectrum was recorded at the light region of **e**.

The pristine Co_3_O_4_ nanocubes prepared by using a surfactant-free method, in which Co_3_O_4_ nanocubes was formed through a reaction of cobalt hydroxides and dissolved oxygen in an aqueous medium. Sequentially a thin (∼1 nm) CoO film was prepared on the single-crystal Co_3_O_4_ nanocube surface by using reducing agent (sodium borohydride) with an accurate control of CoO covering to form a Co_3_O_4_–CoO core–shell nanocube, with the average size of the Co_3_O_4_@CoO single-crystal cubes (SCs) <50 nm (Fig. 1a). A high-resolution transmission electron microscope (HRTEM) was employed to characterize the nanostructure of the core–shell cubes (Fig. 1b; Supplementary Fig. 1) and the selected area electron diffraction of the individual nanocube was characteristic of the single-crystal structure. A set of spots were indexed to the Co_3_O_4_ spinel structure along the [001] zone axis, and the boundary planes of the nanocube were {100}, {010} and {001} (Fig. 1c). A thin, bright layer with a thickness of ∼1 nm was located on the surface of the Co_3_O_4_, indicating that CoO uniformly covered the entire surface of the Co_3_O_4_ nanocube. Corresponding spatial distributions of the compositional elements within the individual nanocube were obtained using a scanning transmission electron microscope–energy-dispersive X-ray line scan through the entire nanocube (indicated in Fig. 1d), revealing that the core–shell nanocube comprised Co and O, and the compositional profiles were symmetrical because of the uniform covering of cobalt oxide. The atomic lattice fringes identified in the HRTEM image was shown in Fig. 1e. It indicated that the nanocube was single crystalline, which was consistent with the result from selected area electron diffraction image. The lattice fringe of a representative nanocube with a *d*-spacing of 2.8 Å could be assigned to the (220) plane of spinel Co_3_O_4_ (JCPDS file nos. 43–1003, *a*=8.084 Å; Supplementary Fig. 2). It was worth noting that the bright outer layer exhibited a clear lattice fringe with a *d*-spacing of 2.1 Å, corresponding to the (200) plane of CoO (JCPDS file nos. 78–0431, *a*=4.2667 Å). To further characterize the thin cobalt oxide layer, electron energy-loss spectroscopy (EELS), a powerful technique for local structural analysis featuring nanometre spatial resolution, was performed. The *L*_3_/*L*_2_ line ratio for the Co-*L* edge served as a fingerprint for the Co valence state^36^, and a 3.2 *L*_3_/*L*_2_ line ratio for the Co EELS peaks was recorded at the middle of the core–shell nanocube as shown in Fig. 1f. This ratio was consistent with the value regarding Co_3_O_4_ (II+III; upper part of Fig. 1f). In the outermost region (lower part), the *L*_3_/*L*_2_ line ratio for the Co peaks was ∼4.5, which is close to the expected value for CoO (II). Consequently, a single-crystal Co_3_O_4_ nanocube with CoO (∼1 nm) uniformly covering the surface was characterized, in which nanocube catalysts exposed the energetically preferred (001) surfaces of OER to electrolyte^32^.

### *In situ* X-ray spectroscopy studies in liquid environment

**Figure 2:**
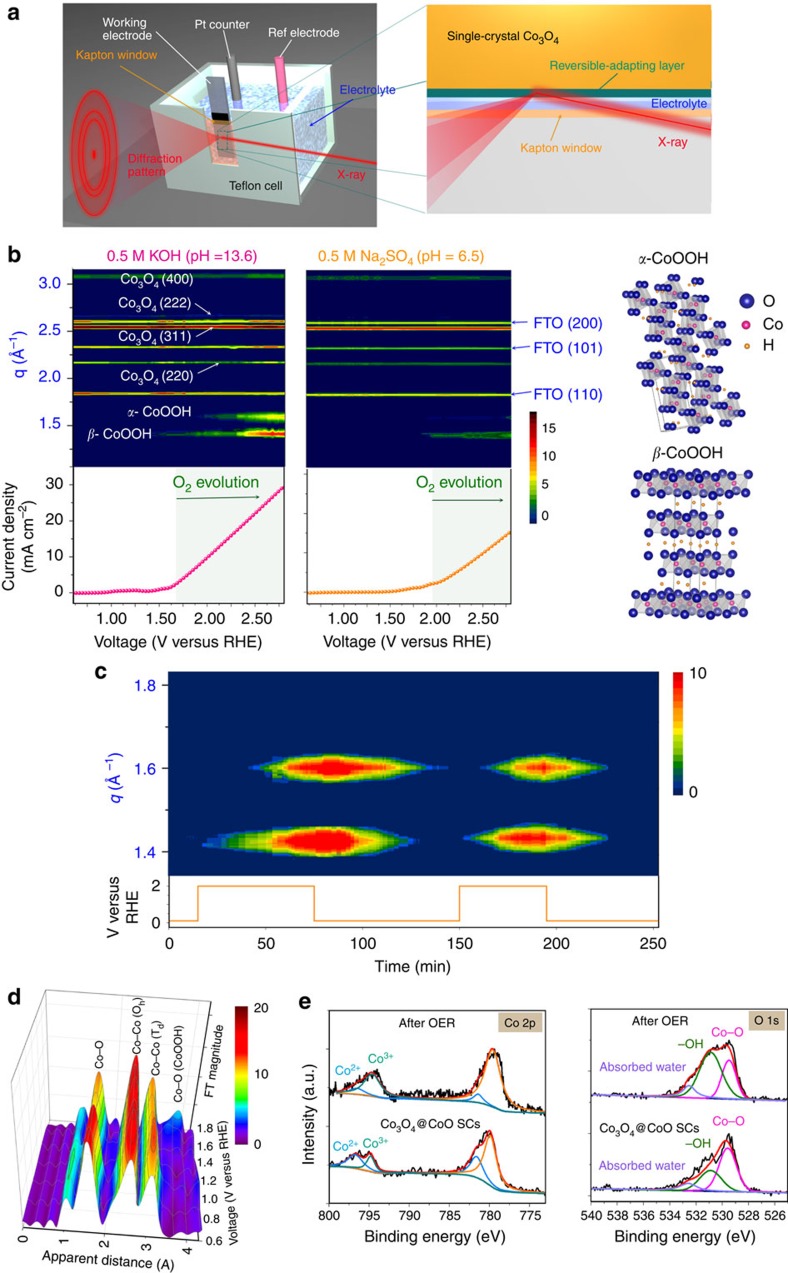
***In situ*** **investigation of cobalt oxide SC catalysts during a water oxidation reaction in a liquid environment.** (**a**) Schematic representation of the *in situ* grazing-angle X-ray diffraction apparatus applied to a liquid electrochemical cell. (**b**) Contour plots of *in situ* grazing-angle X-ray diffraction signals of a Co_3_O_4_@CoO SC in an aqueous solution containing 0.5 M KOH (pH=13.6) and 0.5 M Na_2_SO_4_ (pH=6.5). The images show the diffraction intensity (colour coded) as a function of voltage, and data collection was performed at the NSRRC synchrotron facility by using 12 keV of energy. The lower curves show the measured current density in both cases. (**c**) Contour plots of *in situ* grazing-angle X-ray diffraction signals of a Co_3_O_4_@CoO SC in an alkaline aqueous solution under switching of voltage (between 2.0 and 0.1 V versus RHE), and data collection was performed at the synchrotron facility by using 10 keV of energy to achieve better position distinguishability. (**d**) Voltage-dependence Fourier transform extended X-ray absorption fine structure spectra of a Co_3_O_4_@CoO SC in an alkaline aqueous solution containing 0.5 M KOH (pH=13.6) and in an *in situ* liquid cell. (**e**) XPS Co 2*p* and O 1*s* core-level spectra of the Co_3_O_4_@CoO SCs before and after oxygen evolution reaction.

Since the catalytic performance is strongly dependent on the relevant surface state of electrocatalysts, it would be highly desirable if surface reactive structures could be monitored in liquid condition. In gas evolution situation, obtaining stable signals and monitoring the surface state of catalysts in liquid environment is an extremely difficult task. To achieve this aim, we customized a reaction cell and utilized the synchrotron radiation light source owing to the extremely high intensity and flux of the light source. An *in situ* grazing-angle X-ray diffraction approach in liquid environment can be achieved to determine the atomic structure of the catalysts surface in ambient condition (Fig. 2a), especially for gas evolution situation (high potential region). Figure 2b depicts the current density as a function of applied voltage, and are colour coded together with diffracted intensities recorded using 12 keV of synchrotron light. At the early stage (before O_2_ evolution), main reflexes were attributed to the fluorine-doped tin oxide substrate and the spinel-type Co_3_O_4_ phases. No CoO phase was observed, indicating that the CoO layer was too thin to diffract the light source (a few atomic layers). At a higher voltage, accompanying the formation of *β*-CoOOH (JCPDS file no. 14-0673) on the surface of electrode, the electrodes began oxygen gas evolution in both alkaline and neutral conditions. This suggested that the CoO layer was converted into more active phase with increasing applied potential. When the applied voltage was further increased, a new phase, *α*-CoOOH (JCPDS file no. 26-0480) was observed. Both alkaline and neutral conditions demonstrated a similar phenomenon that OER was strongly correlative with the formation of metal oxyhydroxide. This was direct evidence for the formation of metal oxyhydroxide during higher oxygen gas evolution (∼30 mA cm^−2^) in an *in situ* liquid environment. Notably, the CoO layer functioned as an adapting junction between electrolyte and catalyst to ease off the strain in the nanocrystal substrates when the active phases were formed. As shown in Fig. 2c, when applied potential was cycled between +2.0 and +0.1 V (versus RHE; Reversible Hydrogen Electrode), the *in situ* X-ray diffraction approach showed that this junction layer could alter its structure reversibly between metal oxyhydroxide and amorphous phases. This observation revealed that this skin junction could reversibly adapt to the environment (applied potential) and condition change during catalyzing reaction, consequently protecting the underlay catalyst (for example, Co_3_O_4_) anodizing from applied bias. Moreover, we performed *in situ* X-ray absorption in a liquid environment in low throughput current (below +1.7 V versus RHE) by using the setup shown in Supplementary Fig. 3, and the extended X-ray absorption fine structure spectra of Co_3_O_4_@CoO SCs were depicted in Fig. 2d. We attributed the first peak at ∼1.5 Å and the second and third peaks at ∼2.5 and 3.1 Å, respectively, to the single scattering paths of the closest oxygen (that is, Co–O) and the second/third neighbouring cobalt metals (that is, Co–Co) surrounding the absorbing Co atoms. The Co–O distance (∼1.5 Å) was typical for a Co_3_O_4_ normal spinel-type structure, and the first Co–Co distance (2.5 Å) was typical for a Co (III)–Co bond with octahedrally coordinated Co atoms in a normal spinel structure. The second distance of 3.1 Å was attributed to Co (II)–Co bonds with tetrahedrally coordinated Co atoms. These results were consistent with the results determined for the spinel structure of Co_3_O_4_. Notably, a new peak was observed at ∼3.8 Å when a higher potential of +1.7 V (versus RHE) was applied to a Co_3_O_4_@CoO SC electrode. The peak was attributed to the formation of cobalt oxyhydroxide phase on the surface of the Co_3_O_4_ SC thus led to a new scattering path of Co–O from the cobalt oxyhydroxide phase, which was consistent with the results from X-ray photoelectron spectroscopy (Fig. 2e) and further confirmed the observation in our X-ray diffraction experiments. It was noted that the CoOOH layer formed from Co_3_O_4_ nanocubes without covering of CoO skin was characteristic of rough nature. Unlike Co_3_O_4_ nanocubes, the CoOOH layer formed from Co_3_O_4_ nanocubes with covering of CoO skin exhibited a continuous and uniform surface. This observation can be attributed to the presence of CoO skin layer, while directly anodizing of Co ions on Co_3_O_4_ nanocubes surface resulted in a remarkably rough layer (Supplementary Fig. 4). Using the voltage-dependent *in situ* X-ray diffraction/absorption approach, we concluded that a strong correlation exists between O_2_ gas evolution and the active metal centres with oxyhydroxide phase. More importantly, this junction layer can reversibly adapt its structure to accommodate harsh condition/environment without breaking the underlying scaffold.

### Electrochemical performance

**Figure 3:**
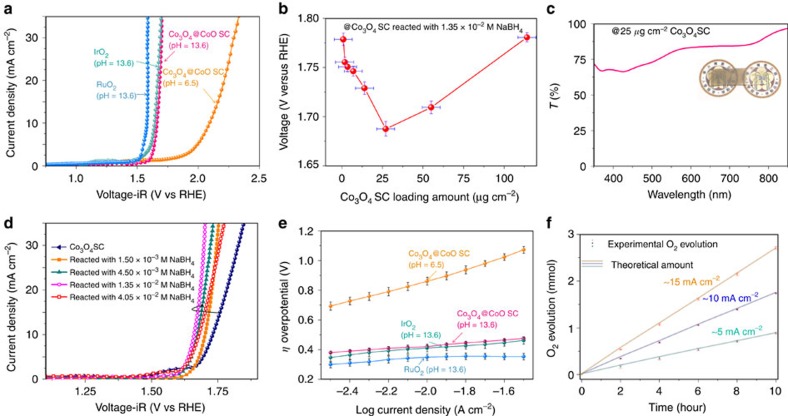
Electrochemical characterization of Co_3_O_4_@CoO single-crystal nanocubes. (**a**) OER polarization curves of Co_3_O_4_@CoO single-crystal nanocubes in alkaline (0.5 M KOH; pH ∼13.6) and neutral (0.5 M Na_2_SO_4_; pH ∼6.5) conditions, commercial RuO_2_ catalysts (0.5 M KOH; pH ∼13.6), and IrO_2_ catalysts (0.5 M KOH; pH ∼13.6); the voltage was corrected according to iR loss from solution resistance. All measurements were performed using an identical catalyst amount (25 μg cm^−2^) and rotating disk electrode (RDE) voltammetry upon a glassy carbon (GC) disk. (**b**) Voltage (versus RHE@ 5 mA cm^−2^) as a function of the catalyst loading amount (μg cm^−2^), indicating that applying the optimized loading amount (25 μg cm^−2^) facilitated achieving the optimal overpotential value for the OER. The error bars are defined as s.d. (**c**) Transmission spectrum of a Co_3_O_4_@CoO SC electrode at a loading amount of 25 μg cm^−2^. Inset: digital photographs of a Co_3_O_4_@CoO SC electrode. (**d**) Polarization curves for the OER of Co_3_O_4_@CoO single-crystal nanocubes prepared using various reductant (NaBH_4_) concentrations in O_2_-saturated KOH (0.5 M; pH ∼13.6) and at a 10 mV s^−1^ scan rate. (**e**) Steady-state Tafel (overpotential versus log current) measurements of Co_3_O_4_@CoO single-crystal nanocubes in alkaline and neutral conditions, commercial RuO_2_ catalysts (0.5 M KOH; pH ∼13.6), and IrO_2_ catalysts (0.5 M KOH; pH ∼13.6). All measurements were performed using RDE voltammetry upon a GC disk in desired electrolyte. Each data point represents the average of ten individual electrode samples where the error bars are defined as s.d. (**f**) Comparison of the O_2_ gas evolution and the charge throughout the circuit (line) in various current densities where the error bar are defined as s.d., indicating that the Faradaic efficiency of water oxidation was close to 100% with no substantial loss.

We evaluated the electrochemical activity of the Co_3_O_4_@CoO SCs during the OER in alkaline and neutral conditions while IrO_2_ (Supplementary Fig. 5) and RuO_2_ (Supplementary Fig. 6) catalysts were also tested as references (Fig. 3a). The ohmic potential drop loss caused by the solution resistance was corrected. Noted that the loading amount of the Co_3_O_4_@CoO SCs was optimized to attain the optimal conditions (∼25 μg cm^−2^; Fig. 3b), in which the catalysts layer could provide a wide range of transparency in the visible region (Fig. 3c). This could allow the development of an electrocatalytic system without inner-filter effects that led to the absorption of an excessive amount of incident light and hindered charge transfer across the semiconductor/electrolyte interface^37^. Both RuO_2_ and IrO_2_ catalysts exhibited slightly lower onset potentials (1.56 V versus RHE@ 5 mA cm^−2^ and 1.61 V versus RHE@ 5 mA cm^−2^) as compared with that of Co_3_O_4_@CoO SCs, presumably because of the intrinsic nature of RuO_2_ and IrO_2_ catalysts^18^,^19^,^38^. Furthermore, the Co_3_O_4_@CoO SC could be operated in a neutral environment as well, except the OER onset potential observed shifted towards a higher voltage. This was attributed to the formation of more active phase on the surface of the Co_3_O_4_@CoO SC in the alkaline condition and was consistent with theoretical and experimental Pourbaix diagrams^31^,^33^. It was noted that the thickness of the CoO layer should be tuned to achieve optimal condition since the usage of a larger amount of reductant led to some undesired reactions on Co_3_O_4_ SC instead of further increasing thickness of CoO layer and resulted in a negative effect in their electrochemical performance (Fig. 3d).

The steady-state Tafel measurements of Co_3_O_4_@CoO SC, IrO_2_ and RuO_2_ were shown in Fig. 3e and Table 1. The overpotential (*η*) comparison of various electrocatalysts to reach a current density of 10 mA cm^−2^ was promising because this value was the approximate current density necessary for 10% efficient solar-to-fuel conversion^19^,^39^. The Co_3_O_4_@CoO SC in alkaline condition yielded a current density of 10 mA cm^−2^ at a low overpotential of 0.430 V, which was similar to the *η* requirement for IrO_2_ (0.411 V) and RuO_2_ (0.358 V) identified both in the present study and relevant literatures^18^,^19^. The Tafel slopes of Co_3_O_4_@CoO SC were unable to achieve the lowest value that compared with those of other 3*d*-transition metal oxide systems^18^,^19^,^20^,^26^. We remarked that a low electrocatalyst loading amount (25 μg cm^−2^) was employed here, so that the catalysts can potentially be used in a photoelectrochemical system. Because of the low loading of electrocatalysts, the mass activity and the turnover frequency (TOF) calculated using these data achieved a mass activity of ∼234.0 A g^−1^ at *η*=0.4 V, which is equivalent to a TOF of ∼0.0487, s^−1^; this value is higher than that of other cobalt-based oxide systems to which high loading amounts were applied (approximately mg cm^−2^), despite that these values were determined in slightly dissimilar conditions and were not directly comparable^11^,^18^,^40^,^41^. Comparing the quantities of gases produced and the amount of charge that passed through the circuit revealed that the Co_3_O_4_@CoO SCs exhibited nearly 100% charge-to-chemical Faradic efficiency during 10 h in various current densities, indicating that these electrocatalysts could enable efficient carrier transportation without noticeable energy loss (Fig. 3f).

### Stability studies of single-crystal electrocatalysts

**Figure 4:**
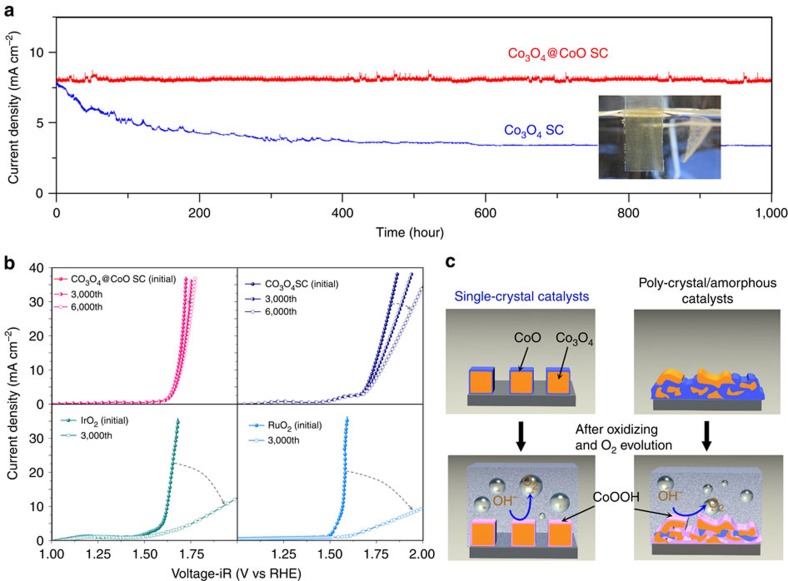
Stability test. (**a**) Current versus time data on a Co_3_O_4_@CoO SC electrode and Co_3_O_4_ SC electrode in 0.5 M KOH (pH=13.6) for 1,000 h (>40 days) at a constant potential of 1.85 V (versus RHE), in which no significant current throughput decay was observed as compared to Co_3_O_4_ SC electrode. (**b**) OER polarization curves of Co_3_O_4_@CoO SC, Co_3_O_4_ SC, IrO_2_, and RuO_2_ catalysts subjected to continual potential cycling between 1.0 and 2.0 V (versus RHE) in O_2_-saturated KOH (0.5 M) for 3,000 (and 6,000) cycle measurements; the Co_3_O_4_@CoO SC retained activity, unlike the IrO_2_ and RuO_2_ control catalysts, which failed to achieve the initial current density. All measurements were performed using rotating disk electrode (RDE) voltammetry upon a glassy carbon (GC) disk in desired electrolyte. (**c**) Schematic representation of structural transformation within single-crystal and amorphous/polycrystal electrocatalysts, indicating that the formation of more active/oxidized phases may generate structural stress and/or voids and weaken the stability of materials. The CoO layer that eliminated the substantial volume change during the OER provided excellent protection for the Co_3_O_4_ nanocubes.

Prolonged testing of the optimized Co_3_O_4_@CoO SCs at a current density of 8 mA cm^−2^ was performed to maintain a constant current density for longer than 1,000 h (>40 days; Fig. 4a and Supplementary Movie 1) without noticeable decay in the current or detaching from the substrate (measured using SEM; Supplementary Figs 7,8 and 9). In contrast, the Co_3_O_4_ SCs was unable to achieve a stable current output, showing a remarkable decrease by 55% after 1,000 h of operation. This result suggested that the reliability of the Co_3_O_4_@CoO SC catalyst was superior to all previously reported systems; even after 1,000 h of operation, gas bubbles still emanated from the electrode surface (Supplementary Movie 1). To further test the long-term stability, we subjected it to a continual potential cycling between 1.0 and 2.0 V (versus RHE) and used the IrO_2_ and RuO_2_ catalysts as controls in O_2_-saturated 0.5 M KOH while all measurements were performed using rotating disk electrode (RDE) voltammetry upon a glassy carbon (GC) disk in the desired electrolyte. The conditions in the test were severe upon OER, because the potential was alternately applied, and the structure of the catalyst could be damaged by the continual valence cycling. Nevertheless, after 3,000 cycles of measurement, the Co_3_O_4_@CoO SCs exhibited no significant decay in its activity, unlike the IrO_2_ and RuO_2_ catalysts, which exhibited rapid decay in performance and decreased to a negligible level (3.1 and 2.6 mA cm^−2^ for IrO_2_ and RuO_2_ at 1.68V versus RHE , respectively) at the 3,000th scan (Fig. 4b). The bare Co_3_O_4_ SCs could only retain 54% of the initial current density after 6,000 scans (at 1.78V versus RHE) while the activity of Co_3_O_4_@CoO SCs did not show noticeable decay. Our results suggested that incorporation of a reversible adapting layer covering nanocrystal electrocatalyst could become an effective design strategy for improving the reliability of electrocatalysts. The spinel type is the most stable phase in Co-based system^18^,^31^, and it is able to transform into active oxyhydroxide phase as well. Unlike spinel-type Co_3_O_4_, a rock salt CoO layer is kinetically more facile to achieve the structural transformation into active oxyhydroxide phase in surface metal centres. This was indicative that the linear sweep voltammetry curve of the Co_3_O_4_@CoO SCs electrode exhibited a considerably lower OER onset potential and higher current density compared with those of the bare Co_3_O_4_ SC electrode (Fig. 3d).

## Discussion

Clearly, the employment of rock salt CoO layer can mediate the interface of electrolyte/electrocatalysts and play multiple roles for efficiently catalyzing the oxidation of water. First, the surface CoO layer is kinetically facile to reach the active oxyhydroxide phase in active metal centres and serve as the active skin for OER. Active metal centres with oxyhydroxide phases are formed *in situ* with lower overpotential to facilitate efficient oxygen evolution reactions. Second, the CoO film provided an excellent protection to Co_3_O_4_ single-crystal nanocubes, because the volume change from Co anodization can be buffered by the CoO layer to avoid mechanical damage to the underlay Co_3_O_4_ scaffolds. The Co_3_O_4_ nanocubes (∼22 Å^3^ per Co) need to undergo a drastic volume change in order to transform into the active metal oxyhydroxides. However, the phase transformations from Co_3_O_4_ to *β*-CoOOH (∼30 Å^3^ per Co) and/or *α*-CoOOH (∼29 Å^3^ per Co) caused drastic volume expansions of approximately 36 and 32%, respectively. Therefore, the surface CoO layer can effectively buffer the harsh structural variations without damaging the Co_3_O_4_ scaffold. Third, single-crystal Co_3_O_4_ nanocubes are characteristically defect free in nature, and thus they avoid self-oxidizing and offer a robust scaffold without energetic losses. In contrast, in the case of amorphous/polycrystal, the formation of more active/oxidized phases are launched from grain boundaries and/or the structural defects, which invariably generates structural stress and/or voids. On the contrary, the volume expansion/contraction did not harm the scaffold (Co_3_O_4_) of electrocatalyst owing to the presence of the CoO layer (Fig. 4c). This strategy significantly improves the reliability of an electrocatalyst through the employment of a reversible adapting junction, and should be generalized and is potentially applicable to various electrocatalysis systems. The main guidelines are that this junction should be able to produce the catalytic metal centre with active phase, withstand the volume change from reaction and mediate the interface of catalyst/electrolyte.

In conclusion, the developed single-crystal Co_3_O_4_ nanocube catalysts with reversible adapting CoO skin layers are reliable, composed of common earth materials, environmental friendly and can be used as efficient water oxidation catalysts that can be incorporated into electrochemical/photoelectrochemical systems. An *in situ* X-ray diffraction method was used to identify a strong correlation between the O_2_ gas evolution and the formation of active metal centres with a metal oxyhydroxide phase through skin layer in liquid condition, and further revealed that this skin layer could remarkably protect underlay electrocatalysts through reversible transformation to active phases. Thus, reversible adapting CoO skin layers and single-crystal Co_3_O_4_ nanocubes play complementary roles to achieve an active and robust electrocatalyst, and lead to retaining a stable current for >1,000 h without exhibiting noticeable decay. These results provided significant progress towards the understanding of the phase of active metal centres on the electrocatalysts, and the advance of the reliability of OER electrocatalysts. This *in situ* X-ray technique provides a powerful tool to the investigation of the phase of surface active metal centre in electrocatalysis and potentially can be applied in probing other catalysis systems. In practical application, solar water splitting or artificial photosynthesis device can be achieved through integration of a reliable catalytic system with photovoltaic solar cell, which captures light energy to generate sufficient driving force for H_2_/O_2_ generation. For instance, Luo *et al.*^42^ recently demonstrated a perovskite tandem solar cell with incorporation of metal layered double hydroxide catalyst to realize a solar-to-hydrogen efficiency of 12.3% for water photolysis. The remarks described here can serve as a new strategy to design robust electrocatalysts for artificial photosynthesis/water photolysis that allows for the conversion of sunlight into O_2_ and chemical fuels, where low catalyst loading is essential for the photoelectrochemical system. Although a cobalt-based material was used as a platform to examine the single-crystal electrocatalysts in this study, the performance of the single Co compounds can be greatly optimized by further incorporating other functional materials. Our strategy here provided a fundamentally design of promising electrocatalysts and should become an accepted technique for electrochemical/photoelectrochemical systems.

## Methods

### Materials

Sodium borohydrate (NaBH_4_) and sodium nitrate (NaNO_3_) were purchased from Acros Organics. Cobalt nitrate hexahydrate, potassium hydroxide and sodium sulfate were obtained from Sigma-Aldrich. Sodium hydroxide was purchased from Fluka. All chemicals were used without further purification. The water used throughout this investigation was reagent-grade water produced using an ELGA ultrapure-water purification system (18.2 MΩ cm).

### Preparation of single-crystal Co_3_O_4_@CoO nanocubes

To synthesize the Co_3_O_4_ nanocubes, NaNO_3_ (45.0 g) was dissolved in an aqueous solution of 0.3 M NaOH (100 ml) in a 3-necked round bottom flask at 100 °C and stirred vigorously while the temperature was maintained for 5 min. After the mixture was stirred for 5 min, an aqueous solution containing 1.0 M Co(NO_3_)_2_ (20 ml) was quickly added to the flask and heated to 115 °C in purified air (50 ml min^−1^). The colour of the precipitate slowly changed from purple to brown and finally to black after being heated for 12 h. The resulting black colloid (Co_3_O_4_ single-crystal nanocubes) was centrifuged at 7,000 r.p.m. and then washed several times with HCl (1.0 M) and deionized water. To prepare the thin CoO layer on the surface of the as-prepared Co_3_O_4_ single-crystal nanocubes, 300 μl of the Co_3_O_4_ nanocube colloid (0.19 g l^−1^) was reacted with a desired concentration (1.50–40.5 mM) of a NaBH_4_ aqueous solution (20 ml) during magnetic stirring at room temperature. After 20 min, the solid product was washed several times, separated from the solution through centrifugation and then dried.

### Characterization

The morphology of the specimens was studied by employing TEM using a JEOL JEM-2100F microscope that was operated at 200 kV, a Hitachi H-7650 that was operated at 120 kV, and a JEOL JSM-7600F field emission scanning electron microscope. The specimens were obtained by placing several drops of the colloidal solution onto a Formvar-covered copper grid and evaporating them in air at room temperature. The chemical composition analysis of the single-crystal nanocubes was performed using an energy-dispersive X-ray analysis (EDX) line scan and EELS in a JEOL JEM-2100F FE-TEM that was equipped with an energy-dispersive spectroscopy probe. The loading amounts were determined using an inductively coupled plasma-mass spectrometer (Agilent 7700 e). The crystallographic structures of the nanocubes were analysed by employing X-ray powder diffraction by using an X'Pert Pro diffractometer with Cu Kα radiation (*λ*=1.5418 Å). Real-time oxygen gas evolution was measured by a Gas chromatography (Agilent 7890B) with thermal conductivity detector. The absorption spectra of the Co_3_O_4_ electrodes were measured using a UV–visible/near-infrared Spectrophotometer V670 (JASCO International Co., Ltd) with an integrating sphere. X-ray absorption measurements of the synthesized samples were performed using synchrotron radiation at room temperature with a handmade reaction cell that was designed for this *in situ* measurement. Measurements were performed at the Co K-edge (7,709 eV) by using the sample, which was maintained at room temperature, and the 01C1 beamline at the National Synchrotron Radiation Research Center (NSRRC), Taiwan designed for these experiments. X-ray absorption data were analysed according to standard procedures, including pre-edge and post-edge background subtraction, normalization with respect to edge height, and Fourier transformation. X-ray diffraction measurements were conducted using a handmade reaction cell that was designed for this *in situ* measurement; all spectra were collected using the 01C2 beamline at the NSRRC, which had a radiation light source of 12 keV. It was worth noting that closer examination of the catalysts by using X-ray absorption approach was difficult, because gas bubbles were quickly generated from the increasing applied potential, and thus smooth oscillation cannot be achieved through absorption manner.

### *In situ* grazing-angle X-ray diffraction studies

The grazing incident X-ray diffraction of thin film samples were performed at the BL01C2 beamline of the NSRRC in Taiwan, in which the ring of NSRRC was operated at energy 1.5 GeV with a typical current ∼360 mA. Two pairs of slits and one collimator were set up in experimental hutch to provide a collimated beam with dimensions of typical 0.5 × 0.5 mm (H × V) at the sample position. An incident angle about 0.2° was employed in present study, in which the X-ray irradiation can pass through several thousand Angstroms upon surface of sample. This geometry allows us to significantly enhance the X-ray scattering intensity from the surface of sample. The grazing-angle was optimized to probe the interior of the sample surface and achieved the best *S*/*N* ratio for maximizing scattering signal from electrode surface. In present study, the wavelength of the incident X-rays was 1.033210 Å (12 keV) delivered from the 5 T Superconducting Wavelength Shifter and a Si (111) triangular crystal monochromator, which allowed to achieve an optimized condition for distinguishing scattering rings. The diffraction pattern was recorded with a Mar345 imaging plate detector which was located ∼320 mm behind the sample, while the pixel size of image was 100 m with typical exposure duration of 1 min. To achieve a correct condition, the diffraction angles were calibrated according to Bragg positions of CeO_2_ (SRM 674b) standards in desired geometry, and then a program of GSAS II was employed to obtain corresponding one-dimensional powder diffraction profile with cake-type integration. In terms of liquid cell, a kapton polyimide film (DuPont) with a thickness of 0.06 mm served as X-ray window in liquid cell which was mode of Teflon line and the thickness of electrolyte below 1 mm was preciously controlled to suppress the interference from liquid electrode.

### Electrochemical studies

To achieve fair comparison among all samples and well control the geometric area/impedance loss, all measurements were performed at room temperature by using RDE voltammetry upon a GC disk at 1,600 r.p.m., in which rotating disk working electrode made of glassy carbon (GC electrode, 5 mm in diameter, PINE:AFE3T050GC PINE, area: 0.196 cm^2^) connected to a CHI-704E bipotentiostat (CHI Instruments). Uniformly dispersed electrocatalyst slurry was obtained via an ultrasonic dispersal of electrocatalysts in deionized water. In the case of working electrodes of glassy carbon, the GC electrode was initially polished using 0.05 μm alumina powder, and then 15 μl of the electrocatalyst slurry was placed on the working electrode, which was dried in air at 40 °C. Finally, the catalyst-loaded GC electrode was covered with 20 μl of a 1% Nafion solution diluted using isopropyl alcohol and dried in a nitrogen atmosphere. Before the electrochemical measurement, the electrolyte was degassed by bubbling oxygen for at least 30 min to achieve a saturation condition of oxygen gas. In terms of *in situ* experiments, electrodes were prepared by incorporating as-synthesized nanocubes within a well-defined area (2 × 1 cm^2^) onto a fluorine-doped tin oxide-coated glass (CP-Solar, Tec 7) since GC disk is unable to utilize in such experiments, which was washed using acetone, isopropanol and deionized water before use; the nanocube film electrodes were subsequently annealed at 175 °C for 4 h. Electrochemical experiments were performed in a typical three-electrode system controlled using a CHI-704E or CHI-405A potentiostat (CHI Instruments). Linear sweep voltammetry was performed at a scan rate of 100 mV s^−1^. All potentials were referenced to an Hg/HgO reference electrode or Ag/AgCl reference electrode, and Pt was used as the counter electrode in all measurements, in which the electrolyte was either a 0.5 M Na_2_SO_4_ (pH ∼6.5) solution or a 0.5 M KOH (pH ∼13.6) solution. All potentials were adjusted to compensate for the ohmic potential drop losses (*R*_u_) that arose from the solution resistance and calibrated with respect to RHE.


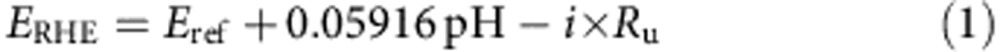


A long-term amperometric *i*–*t* curve was performed using a sampling interval of 1 s, and each run time was 86,400 s (24 h). Regarding the 1,000-h O_2_ evolution, the number of repetitive runs was 42 to ensure that measurement was conducted for 1,000 h. The volume of the electrolyte was kept constant to maintain a constant immersed-area of electrodes.

The values of mass activity (A g^−1^) were calculated using the following equation:


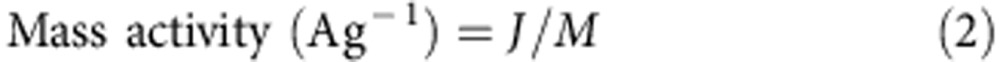


where *M* is the electrocatalyst loading amount (25 μg cm^−2^) and *J* is the measured current density (mA cm^−2^).

The values of TOF were calculated by assuming that every metal atom was involved in the catalysis (lower limits):


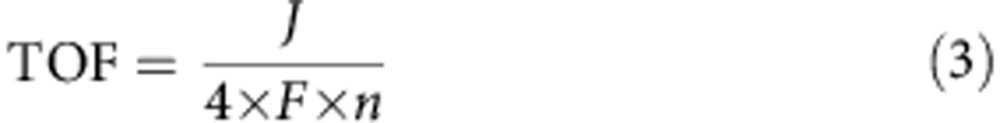


where *J* is the current density (A cm^−2^), *F* is Faraday's constant (96485.3 C mol^−1^) and *n* is moles of electrocatalysts (mol cm^−2^).

## Additional information

**How to cite this article:** Tung, C.-W. *et al.* Reversible adapting layer produces robust single-crystal electrocatalyst for oxygen evolution. *Nat. Commun.* 6:8106 doi: 10.1038/ncomms9106 (2015).

## Supplementary Material

Supplementary Figures Supplementary Figures 1-9

Supplementary Movie 1 This movie file shows oxygen evolution of the optimized Co3O4@CoO single-crystal nanocubes at a current density of 8 mA cm-2.

## Figures and Tables

**Table 1:** Comparison of catalyst activity in the OER* †.

**Catalyst**	***η*****@*****J*****=10 mA cm^−2^** **(mV)**	***J*****@*****η*****=0.4 V (mA cm**^−2^**)**	**Tafel slope (mV dec**^−1^**)**	**Mass activity @*****η*****=0.4 V (A g**^−1^**)**	**TOF @*****η*****=0.4 V (s**^−1^**)**	***J*** **(3,000 th) @*****η*****=0.45 V (mA cm**^−2^**)**
Co_3_O_4_@CoO SC						
(@pH 13.6)	430	5.85	89	234.0	0.0487	12.15
(@pH 6.5)	851	—	375	—	—	—
IrO_2_ (@pH 13.6)	411	9.26	91	370.4	0.2152	3.10
RuO_2_ (@pH 13.6)	358	38.5	55	1,540.0	0.5309	2.63

^*^Dry loading amount: 25 μg cm^−2^.

^†^All measurements were performed using an identical catalyst amount (25 μg cm^−2^) and rotating disk electrode (RDE) voltammetry upon a glassy carbon (GC) disk.
