# Supplementary material for: Reversible adapting layer produces robust single-crystal electrocatalyst for oxygen evolution
Source: Nat Commun. 2015 Aug 28;6:8106. doi: 10.1038/ncomms9106 (PMC4560826; doi:10.1038/ncomms9106)
Supplement: Supplementary Figures — 1-9 [file ncomms9106-s1.pdf]

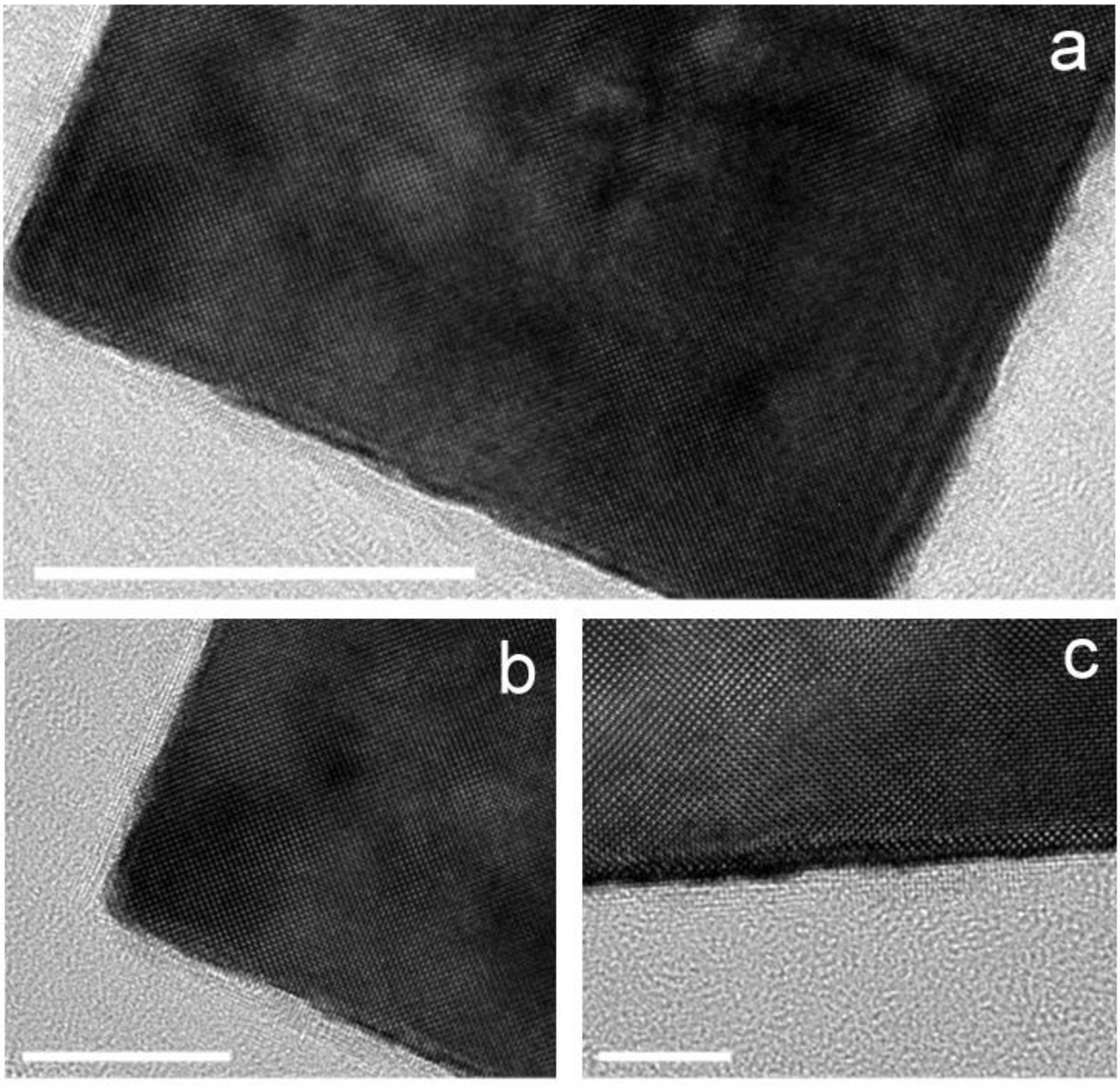

Supplementary Figure 1. Typical HRTEM images of Co_3O_4@CoO single-crystal nanocubes. The dark area is Co_3O_4 and the light area is CoO, indicating that cobalt oxide covered the entire surface of the Co_3O_4 nanocubes. (a) Scale bar is 20 nm. (b) Scale bar is 10 nm. (c) Scale bar is 5 nm.

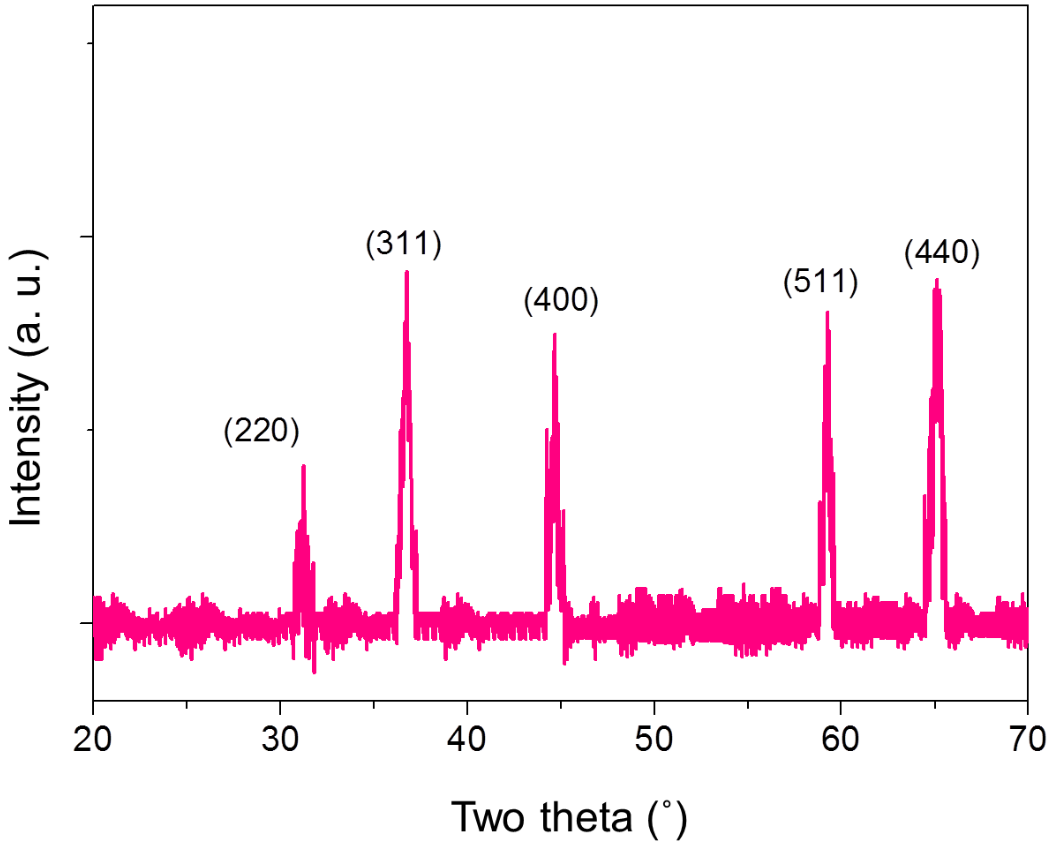

Supplementary Figure 2. X-ray diffraction pattern of as-prepared Co_3O_4 nanocubes. This result was consistent with the result regarding the bulk of the spinel Co_3O_4 structure (JCPDS file nos. 43–1003, a = 8.084 Å).

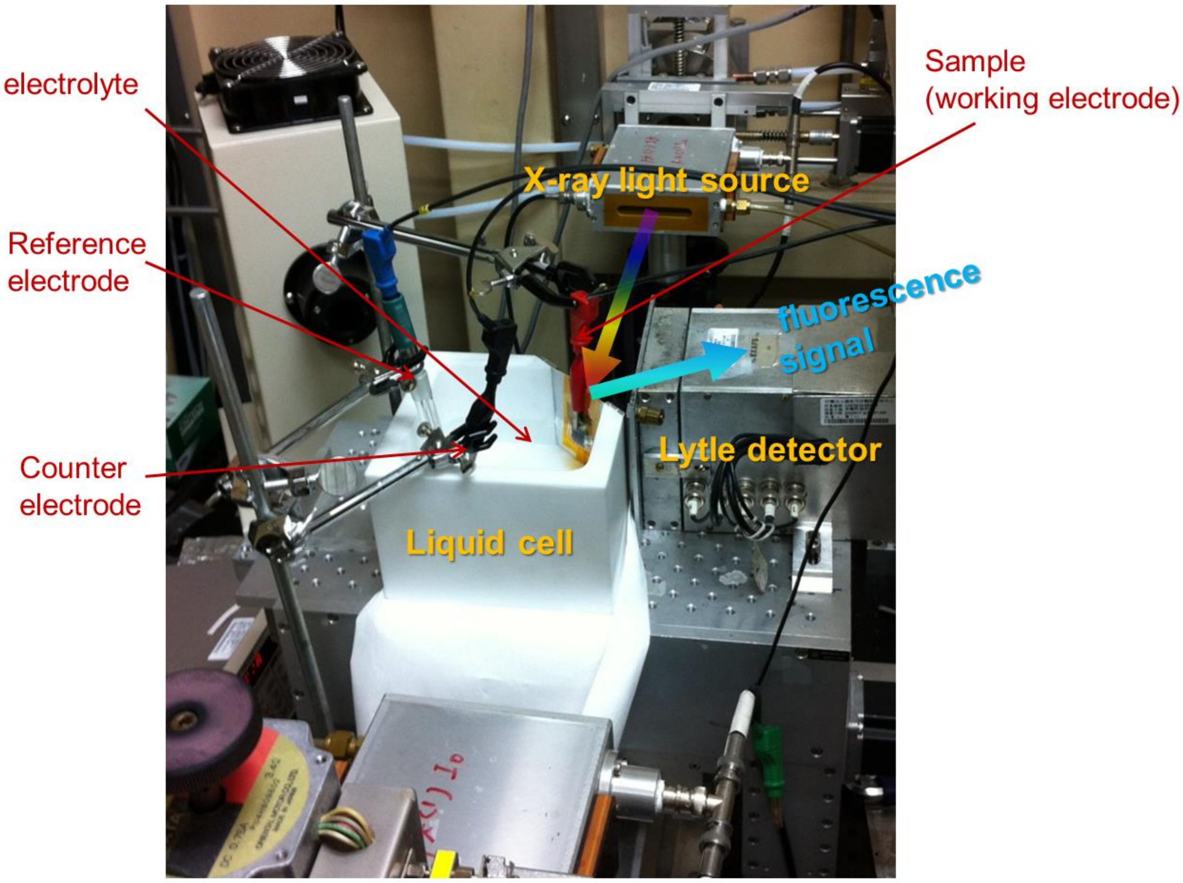

Supplementary Figure 3. Digital photograph of a hand-made chemical cell for use in *in-situ* X-ray absorption measurement. X-ray absorption measurements of the synthesized samples were performed using synchrotron radiation at room temperature with a hand-made reaction cell that was designed for this study. Measurements were conducted at the Co K-edge (7709 eV) by maintaining the sample at room temperature and using the 01C1 beam line at the NSRRC, Taiwan designed for such experiments.

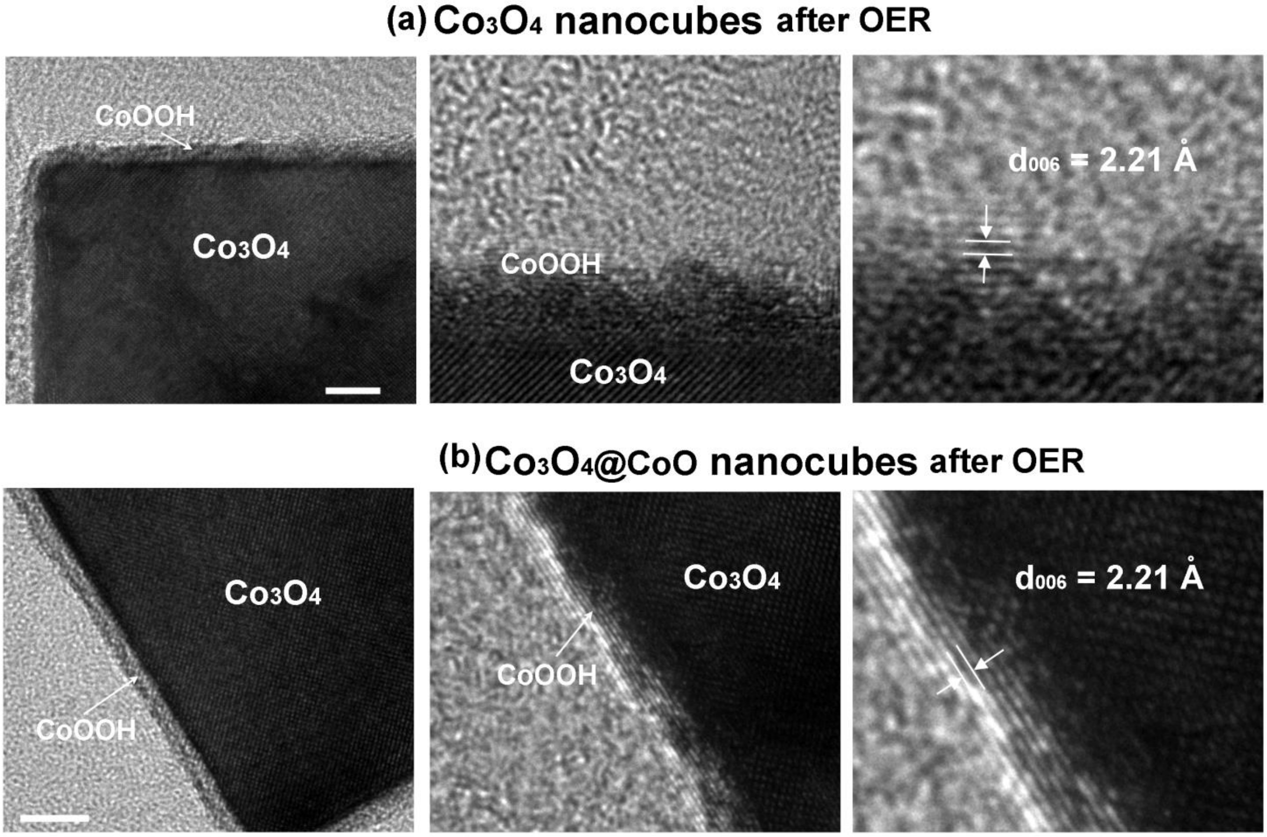

Supplementary Figure 4. High-resolution transmission electron microscopy image of Co_3O_4 nanocubes(a) and Co_3O_4@CoO nanocubes(b) after oxygen evolution for 10 hrs. Scale bars are 5 nm.

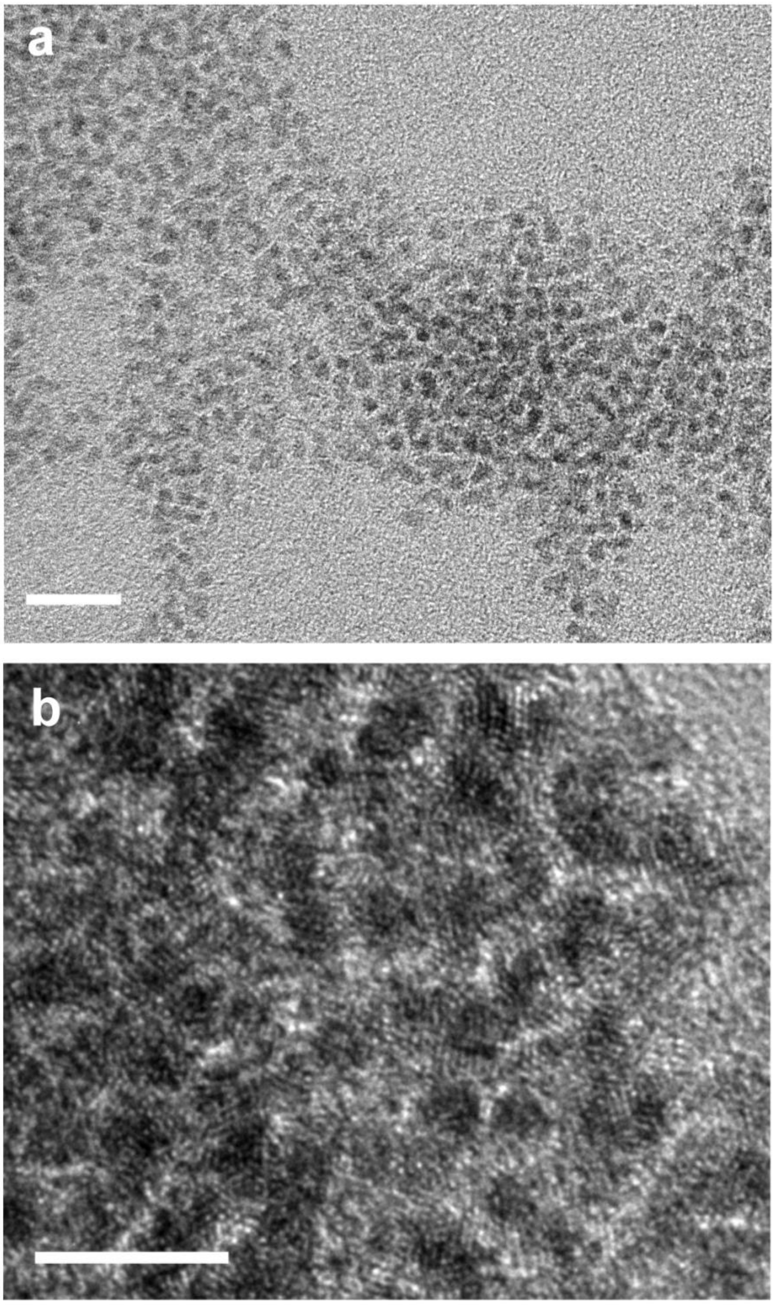

Supplementary Figure 5. Typical transmission electron micrograph and high-resolution transmission electron microscopy image of IrO_2 nanoparticles. Scale bars are (a) 10 nm and (b) 5 nm.

5 nm.

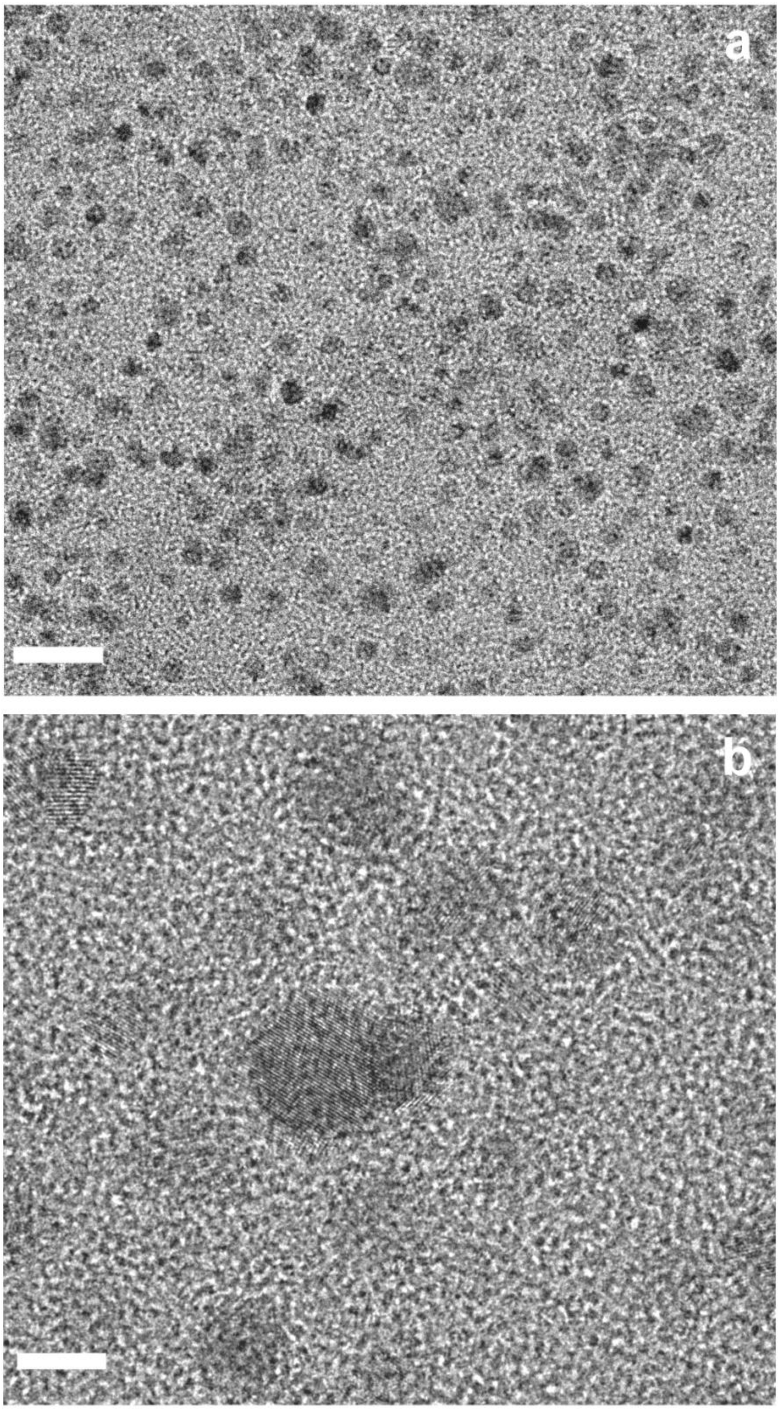

Supplementary Figure 6. Typical transmission electron micrograph and high-resolution transmission electron microscopy image of RuO_2 nanoparticles. Scale bars are (a) 20 nm and (b) 5 nm.

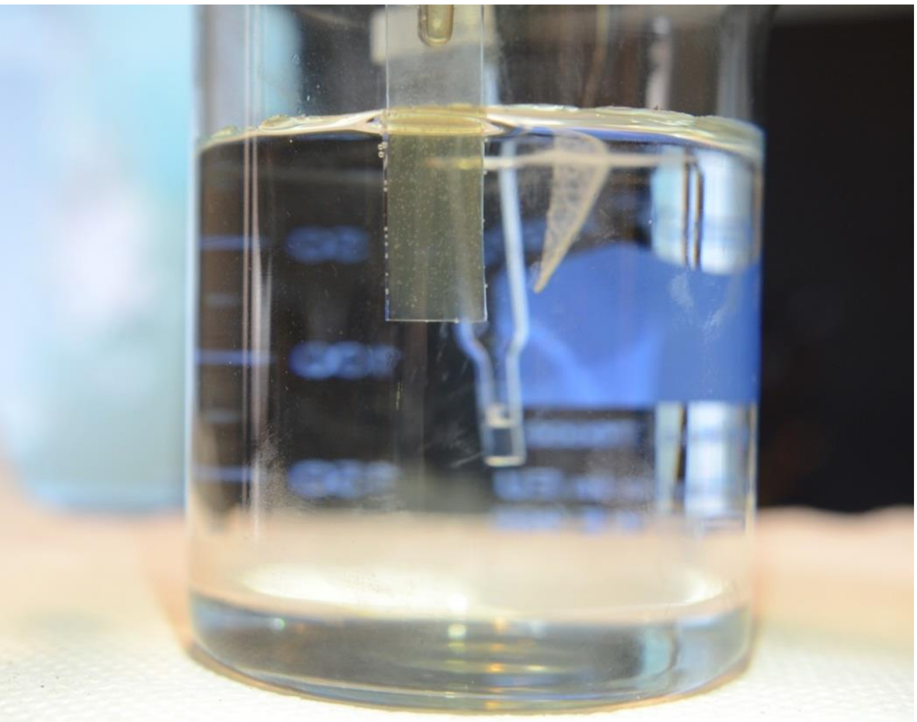

Supplementary Figure 7. Digital picture of a Co_3O_4@CoO SC electrode at a loading amount of 25 μg cm^{-2} under a current density of 8 mA cm^{-2}. This image shows that O_2 bubbles appeared on the surface of the Co_3O_4@CoO SC electrode.

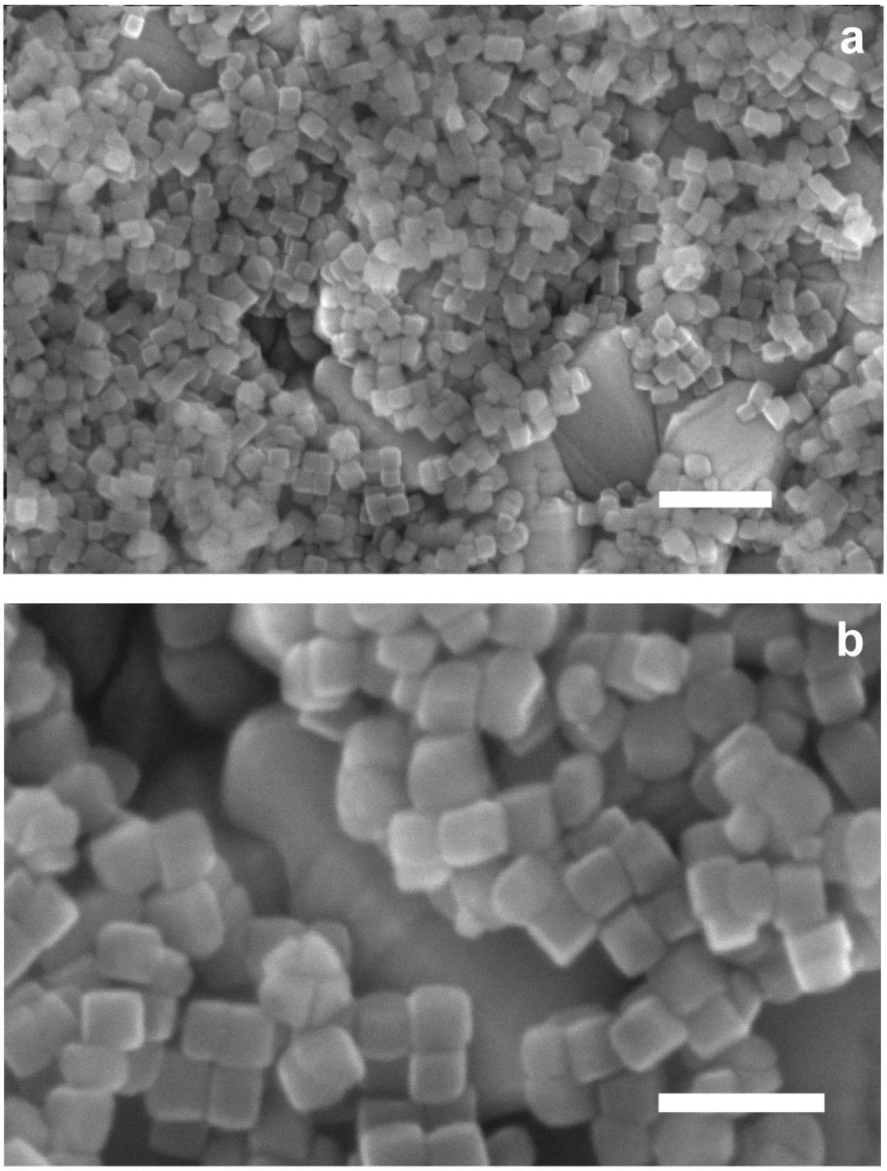

Supplementary Figure 8. Field-emission scanning electron microscope images of a Co_3O_4@CoO SC before oxygen evolution reaction. Scale bars are (a) 200 nm and (b)100 nm.

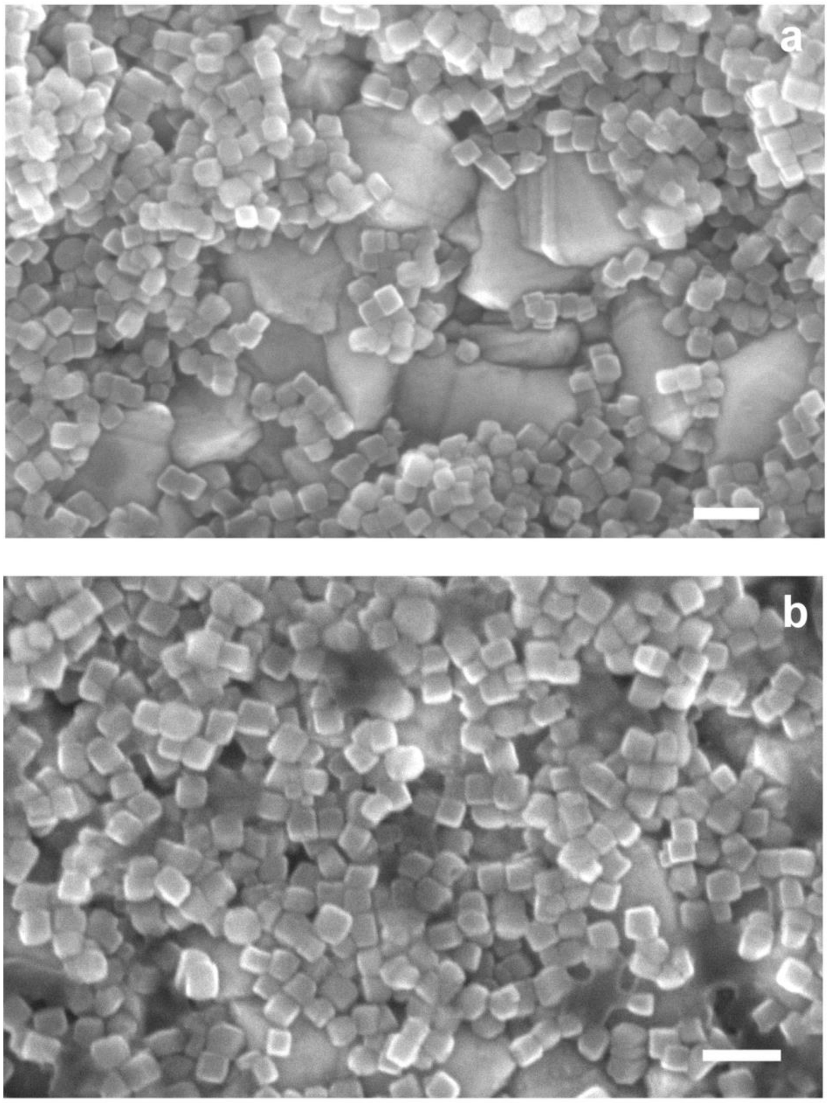

Supplementary Figure 9. Field-emission scanning electron microscope images of a Co_3O_4@CoO SC after the oxygen evolution reaction executed in an aqueous solution containing 0.5 M KOH for 1000 hours. This image shows that the Co_3O_4@CoO single-crystal nanocubes exhibited no noticeable structural change, providing strong evidence that these Co_3O_4@CoO single-crystal nanocubes provide robust and stable performance. The CoO layer provided protection for the Co_3O_4 nanocubes, preventing substantial volume change during the OER, and the single-crystal Co_3O_4 nanocubes served as a robust framework during long-term oxidization. Scale bars are (a) 100 nm and (b) 100 nm.
